# Features of biliary tract diseases in ketamine abusers: a systematic review of case reports

**DOI:** 10.1186/s13256-024-04421-6

**Published:** 2024-03-03

**Authors:** Alireza Teymouri, Hadis Nasoori, Maryamsadat Fakheri, Aref Nasiri

**Affiliations:** 1 Department of Surgery, School of Medicine, Tehran University of Medical Sciences, Tehran, Iran; 2 Faculty of Pharmacy, Tehran Medical Science, Islamic Azad University, Tehran, Iran; 3 Department of Physical Medicine and Rehabilitation, School of Medicine, Shiraz University of Medical Sciences, Zand St., Shiraz, 71348-14336 Iran

**Keywords:** Ketamine, Substance abuse, Biliary tract disease, Cholangiopathy

## Abstract

**Background and aims:**

Anesthesiologists prefer ketamine for certain surgeries due to its effectiveness as a non-competitive inhibitor of the N-methyl-D-aspartate receptor in the brain. Recently, this agent has also shown promise as an antidepressant. However, ketamine can cause hallucinogenic effects and is sometimes abused as an illicit drug. Ketamine abuse has been associated with liver and bile duct complications. This systematic study aims to better understand cholangiopathy in ketamine abusers by reviewing case reports.

**Methods and material:**

In this systematic review, a comprehensive literature search was conducted with the terms “biliary tract diseases” and “ketamine”. Case reports and case series of adult patients with documented ketamine abuse and reported cholangiopathy or biliary tract disease were included. We extracted the data of relevant information and the results were reported through narrative synthesis and descriptive statistics.

**Results:**

A total of 48 studies were initially identified, and 11 studies were finally included in the review. The mean age of the patients was 25.88 years. Of the 17 patients, 64.7% were men. Symptoms often included abdominal pain, nausea, and vomiting. Most patients were discharged with improved symptoms and liver function. Common bile duct dilation and other findings were observed in imaging results and other diagnostic studies.

**Conclusion:**

This review highlights the diverse presentations and diagnostic modalities used in ketamine-induced cholangiography. These patients tend to be young men with deranged liver function tests and abdominal pain, which should be taken into consideration. These patients often require a multidisciplinary approach in their management.

## Introduction

Ketamine is a synthetic phencyclidine derivative with both analgesic and anesthetic properties [1]. It has a molecular weight of 237.72 g/mol and its pKa is about 7.5 [2]. Its onset of action is within 30 seconds of intravenous administration and it has a half-life of 2.5 hours [3]. Ketamine is metabolized by liver through N-demethylation and hydroxylation, which is in turn excreted into urine (90%) and bile (10%) [4]. This substance acts as an antagonist on N-methyl-D-aspartate (NMDA) receptors and noncompetitively blocks glutamate [5].

Anesthesiologists favor ketamine over other agents for certain surgical procedures, and this medication has been widely used for this purpose since its discovery in 1962 [6]. It has also been used as an antidepressant in recent years and has shown promising results [7]. However, ketamine can also exert hallucinogenic effects, vivid imagery, and short-term excitement, and is used as an illicit drug via oral or inhalation routes [8].

Ketamine medical use has been associated with hepatobiliary complications, which is well established in the literature [9, 10]. Burned patients who receive large accumulative doses of ketamine are more prone to develop these complication [11, 12]. Recently, critically ill patients with coronavirus disease 2019 (COVID-19) have also been afflicted with biliary tract diseases such as secondary sclerosing cholangitis [13]. Moreover, similar to burned patients, ketamine abusers who consume large quantities of the drug are susceptible to biliary tract disease [14].

Despite its rarity, there are multiple case reports that address cholangiopathy in ketamine abusers. However, to the best of our knowledge, there is no methodological study that presents the findings of these case reports to give us a better understanding of this complication. Thus, the present systematic review of case reports is designed to better characterize the features of cholangiopathy in ketamine abusers.

## Method

### Literature search and eligibility criteria

**Fig. 1:**
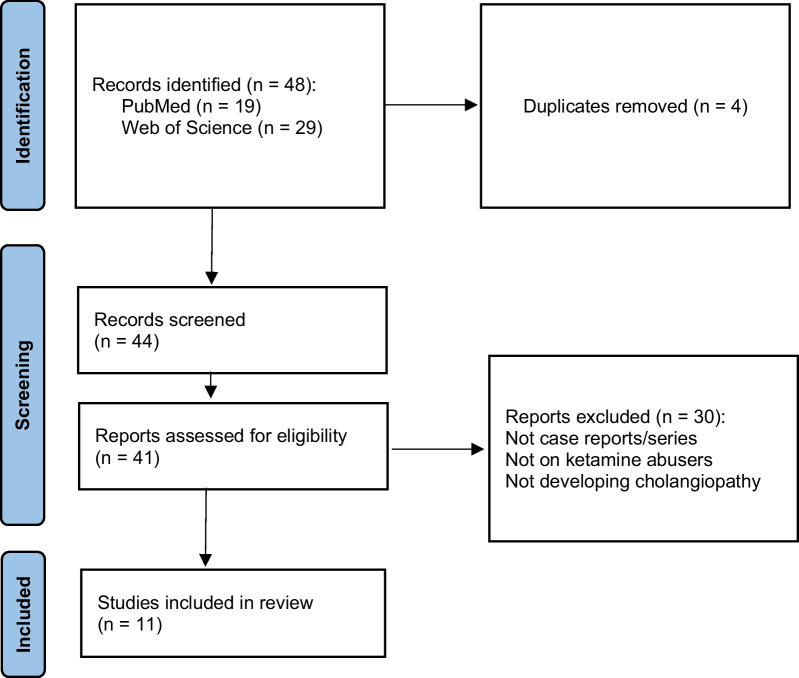
PRISMA flow diagram for study selection

We used the Preferred Reporting Items for Systematic Reviews and Meta-Analysis (PRISMA) guideline to conduct the present systematic review. We carried out a thorough literature search in PubMed, Google Scholar, and Web of Science using the Medical Subject Headings (MeSH) terms “biliary tract diseases” and “ketamine” from inception until October 2023. The full search terms are outlined in the appendix. We did not limit the preliminary search results with any filters. Furthermore, to avoid missing any pertinent article, we hand-searched the references of each selected study. The diagram explaining the selection process is depicted in Fig. 1. Studies were included if they met the following inclusion criteria: (1) were case reports or case series of adult patients aged 18 years or older, (2) patients with documented ketamine abuse, (3) patients with reported cholangiopathy or biliary tract disease, and (4) were written in English language. The following were excluded: (1) non-adult patients, (2) reports with etiologies other than ketamine abuse, such as respiratory distress syndrome, and (3) patients with other confirmed hepatobiliary conditions such as viral hepatitis and cirrhosis.

### Selection of studies

The studies retrieved by the initial search were examined independently by two reviewers (AT and HN). As the first step, we identified the duplicates and removed them. The search results were then truncated to case reports (could be published as letter, correspondence, commentary, and so on) with at least one patient developing cholangiopathy following ketamine administration. Afterward, each paper was evaluated for eligibility on the basis of title, abstract, and full text. If a full-text article was unavailable, the correspondent was contacted for a copy, and in case of disagreement between the reviewers, a decision was made by consensus or by consulting a third reviewer (AN). The selected studies contained information about diagnosis, laboratory tests and imaging, demographics (for example, age and sex), liver function tests, and the outcomes of the disease.

### Data extraction and quality assessment

We extracted the following items after the final selection: study region and year, demographics of patients, past medical history, presenting signs and symptoms, liver function test results, ketamine abuse pattern and duration, imaging findings (for example, ultrasound and magnetic resonance imaging), biopsy and cytology findings, treatment approach, and outcome of the disease (that is,. deceased or discharged). To minimize the effect of biases, we assessed the quality of each included study with a standardized tool [15]. A narrative synthesis was used to report the results and descriptive statistics were used to calculate the frequency and percentage for categorical variables, as well as mean and standard deviation for continuous variables.

## Results

### Study characteristics and patients’ clinical information

Overall, 48 studies (PubMed = 19, Web of Science = 29) were identified through our initial search, of which 4 were duplicates and removed. A total of 33 studies were excluded on the basis of title and abstract, and finally 11 studies were included in this review [16–26]. The study selection process is outlined in Fig. 1. Studies were conducted from 2009 through 2020 in the UK [5], China [2], Hong Kong [3], and the USA [1]. Mean age was 25.88 ± 4.95 years, ranging from 18 to 38 years. Of the 17 patients, 11 (64.7%) were men and 6 were women. We found that three patients were concurrent alcohol abusers and two patients were human immunodeficiency virus (HIV) positive. Urinary tract infection (UTI) was observed in three patients, and two patients had a history of acute renal failure (ARF). A total of 15 patients presented with epigastric or right upper quadrant abdominal pain, along with other signs and symptoms such as nausea, vomiting, and fever. As for the other two patients, one of them was referred because of deranged liver function tests and the other had urinary symptoms. Jaundice and hepatomegaly were detected in one patient.

Liver function tests (LFTs) were not reported for one patient and two patients had normal LFT. The initial alanine aminotransferase (ALT) levels were available for 11 patients, with a calculated mean of 232.09 ± 148.14 IU/L, ranging from 75 to 521 IU/L. These patients had been abusing ketamine for 3 months to 15 years (median 2 years). Of 17 patients, 9 received endoscopic retrograde cholangiopancreatography (ERCP) for treatment, and other treatment approaches included drug rehabilitation program, catheterization, biliary drainage, and conservative therapy. Except for one patient who lacked outcome information, all patients were discharged with improved symptoms and LFT results.

### Imaging results and other diagnostic studies

The main imaging modalities that were used across the studies were ultrasound (US), magnetic resonance cholangiopancreatography (MRCP), computed tomography (CT), and hepatobiliary iminodiacetic acid (HIDA) scan, which were utilized in 13, 9, 10, and 3 patients, respectively. In addition, the findings of ERCP, which is mainly a therapeutic approach, were reported in five patients. Common bile duct (CBD) dilation was visualized in 12 patients. The investigators detected strictures in three patients and gallstone in one patient. HIDA scan showed diminished gall bladder ejection fraction in one patient and was normal in the other two patients. Other diagnostic studies, including brush cytology, colonoscopy, and biopsies, were performed in a subgroup of nine patients. These findings are all summarized in Table 1.

**Table 1:** Summary of study characteristics and patients’ demographic, clinical, and imaging information

No.	Study reference	Age, sex, country	Medical history	Signs and symptoms	LFT	Ketamine abuse pattern and duration	Imaging findings	Other studies	Treatment	Outcome
1	Gutkin *et al*. [1] 2012	18, F, China	Alcohol abuse	N/V, abdominal tenderness	AST 472 IU/L ALT 330 IU/L GGT 226 IU/L	Three times per week for 4 years	US: dilated CBD CT: wall thickening of the gallbladder and dilated common bile duct to 14 mm, without evidence of stones or other obstructing lesions	Cytology: normal. Ampullary biopsy: normal	ERCP: papillotomy	Discharged: normal LFT
2	Gutkin *et al*. [1] 2012	27, M, China	None	N/V, severe abdominal pain, dysuria, abdominal tenderness	Normal	3 g per day doses	US: dilated CBD CT: CBD 7.4 mm MRCP: mild intrahepatic biliary dilatation, moderate extrahepatic dilatation, CBD 11 mm	Cytology: normal. Ampullary biopsy: normal	ERCP: sphincterotomy	Discharged: symptom free after ketamine cessation
3	Turkish *et al*. [2] 2013	21, M, USA	ARF	Fever, abdominal pain, abnormal liver function	Elevated	Daily for 9 months	US: diffusely echogenic liver with normal portal vein blood flow CT: normal MRCP: normal	Liver biopsy: concentric preductal fibrosis consistent with primary or secondary sclerosing cholangitis, with mild lymphocytic infiltrates and a mild ductular reaction, but no cholestasis was identified	Drug rehabilitation program	Discharged: mild elevation of ALP with normal intra- or extra-hepatic bile ducts
4	Lo *et al*. [3] 2011	27, M, UK	ARF	Abdominal pain, tachypnea, tachycardia,	ALT 106 IU/L	For 4 years	US: bilateral hydronephrosis and nephrostomies CT: bilateral hydronephrosis and biliary dilatation Chest radiography: bilateral basal consolidation HIDA: diminished gall bladder ejection fraction	None	Intubation, hemofiltration, and antibiotics, ERCP	Discharged: total resolution of hydronephrosis and the absence of any biliary dilatation
5	Lo *et al*. [3] 2011	27, M, UK	None	Colicky epigastric pain	ALT 75 IU/L	6 mg daily in split doses for 2 years	US: normal Cystoscopy: red, edematous, and ulcerated bladder mucosa HIDA: normal	None	Catheterization	Discharged: lost to follow-up
6	Lo *et al*. [3] 2011	26, M, UK	None	Nocturia, hematuria, and increased urinary frequency	ALT 44–266 IU/L	For 7 years	US: liver and biliary tree were normal Cystoscopy: erythromateous CT and IVU: bilateral hydronephrosis and bilateral hydroureters HIDA: no appreciable filling of the gallbladder	Biopsy: inflammation	Long-term catheterization	Discharged: Gallbladder dyskinesia
7	Wong *et al*. [4] 2009	21, F, Hong Kong	None	Recurrent epigastric pain	ALT 333 IU/L	1 once per 1–2 months for 18 months	US: dilated CBD and normal gallbladder CT: fusiform dilatation CHD and CBD up to 9 mm in diameter MRCP: dilatation of CBD Gastroscopy: mild antral gastritis	None	Biliary drainage	Discharged: MRCP showed resolution of CBD diameter of 4 mm and normal LFT
8	Wong *et al*. [4] 2009	27, M, Hong Kong	Choledochal cyst	Recurrent epigastric pain	ALT 75 IU/L	Twice a week for 2 years	Gastroscopy: mild gastric erosion CT: CBD dilatation up to 17 mm MRCP: Fusiform dilatation of CBD and CHD	Cytology: normal	ERCP: plastic biliary stent	Discharged: decreased CBD diameter and discharged with no pain
9	Wong *et al*. [4] 2009	23, M, Hong Kong	NA	Colicky epigastric pain and ketamine-associated cystitis	Normal	1 once per week for 3 months	CT: fusiform dilatation CHD and CBD up to 11.2 mm in diameter US: N/A	None	Conservative therapy	Discharged
10	Zhou *et al*. [5] 2013	38, M, UK	Well-controlled HIV, asthma and HTN, alcohol abuse	Acute-on-chronic epigastric pain, nausea and vomiting	ALT 131 IU/L	1–2 g a day for a year	Endoscopy and barium studies: normal MRCP: dilatation of CBD, mild cholangiopathy, no gallstones ERCP: no obstructive lesion	Duodenal biopsies: normal	ERCP: sphincterotomy	Discharged: reduction in CBD diameter and normal LFT
11	Zhou *et al*. [5] 2013	25, M, UK	HIV positive, alcohol abuse, UTI	Intermittent right upper quadrant pain and nausea	ALT 418 IU/L GGT 1015 IU/L	1 g 2–3 times per week for 12 months	US: CBD dilation, normal liver and pancreas MRCP: CBD dilatation of 14 mm; normal gallbladder, no intra-ductal stones ERCP: normal	Liver biopsy: a non-cirrhotic liver, no features of HIV cholangitis, opportunistic infections, or alcohol toxicity	ERCP: sphincterotomy	Discharged: pain subsequently resolved and LFTs normalized within 2 months of stopping ketamine
12	Seto *et al*. [6] 2011	32, F, China	UTI	On-and-off epigastric discomfort	GGT 284 U/l	For 7 years	ERCP: multiple long-segment strictures and narrowing in the intrahepatic ducts of both lobes, CBD dilation, pancreatic duct was normal	-Brush cytology: only reactive changes -Liver biopsy: mild nonspecific inflammation of the portal tracts -Colonoscopy with biopsies: normal	ERCP	N/A
13	Cheung *et al*. [7] 2014	20, F, Hong Kong	None	Right upper quadrant pain, nausea and fever	ALT 178 U/L	For 2 years	CT: CBD dilatation, evidence of hepatic micro-abscesses ERCP: persistent ultra-short narrowing was noted at the very distal portion of CBD, where sphincter of Oddi would be located	None	ERCP: sphincterotomy, sphincteoplasty	Discharged
14	Al-Nowfal *et al*. [8] 2016	24, F, UK	None	Intermittent right upper quadrant pain associated with nausea and malaise	NA	For 4 years	US: dilated CBD and normal gallbladder MRCP: no gallstones and normal CBD	None	Opiate analgesics and antispasmodics	Discharged
15	Aslam *et al*. [9] 2019	24, F, UK	UTI	Right upper quadrant pain,	ALT 521 U/L	N/A	MRCP: bilateral ureteric thickening, moderate intrahepatic biliary dilatation and strictures, with thickening of the common duct wall and a stricture of the inferior common duct US: diffuse thickening of the common duct	None	N/A	Discharged: reduction in biliary dilatation and LFT
16	Nyirenda *et al*. [10] 2020	32, M, UK	None	Jaundice, rigors, and decreased appetite, right hypochondrium pain and tender hepatomegaly	ALT 203 U/L AST 86 U/L GGT 2050 U/L	Daily for 15 years	MRCP: dilated bile ducts with no filling defects, gallstones or strictures US: normal CT: normal	None	ERCP: plastic stent	Discharged: improved LFT
17	Lui *et al*. [11] 2014	28, M, Hong Kong	None	Deranged liver function test results	ALT 183 IU/L GGT 1088 IU/L	For 5 years	US: dilated CBD with a gallstone ERCP: 5 cm stricture at the lower end of the CBD together with small bilateral segmental strictures in the intrahepatic ducts	-Brush cytology: normal -Liver biopsy: mild-to-moderate portal fibrosis with ductular proliferation and periportal copper deposits -Colonoscopy: normal	ERCP: plastic stent	Discharged: improved LFT

N/V: nausea or vomiting; AST: aspartate aminotransferase; ALT: alanine transaminase; GGT: gamma-glutamyl transpeptidase; US: ultrasonography; CBD: common bile duct; CT: computed tomography; ERCP: endoscopic retrograde cholangiopancreatography; LFT: liver function test; MRCP: magnetic resonance cholangiopancreatography; ARF: acute renal failure; ALP: alkaline phosphatase; HIDA: hepatobiliary iminodiacetic acid; CHD: common hepatic duct; HTN: hypertension; UTI: urinary tract infection; N/A: not available

## Discussion

This study provides valuable information about the characteristics of patients who presented with ketamine-induced cholangiopathy. The mean age of the patients was 25.88 ± 4.95 years, with a range from 18 to 38 years. Men accounted for 64.7% of the patients, while women accounting for the remaining 35.3%. Additionally, three patients were found to be alcohol abusers and two were HIV positive. Among the patients, three had UTIs and two had a history of ARF. The epidemiology of other biliary diseases, such as primary sclerosing cholangitis with a 2:1 male to female ratio, is comparable to our results [27]. The patients involved in this review were rather young, which can be explained by the recreational nature of the ketamine abuse. Concomitant alcohol abuse is quite common in ketamine abuser population and can be up to 25% [28]. Due to the possible cytotoxic effect of ketamine on urothelium, patients often experience urinary symptoms [29]. This explains the resurgence of urinary symptoms in the patients included in this review.

The most common presenting symptom was epigastric or right upper quadrant abdominal pain, which was reported by 15 patients and also witnessed in clinical practice. Two other patients either presented with deranged liver function tests or urinary symptoms. LFTs were available for all but one patient, with two patients having normal LFT results. The initial ALT was measured in 11 patients, with a mean value of 232.09 ± 148.14 IU/L, ranging from 75 to 521 IU/L. The upper limit of normal value (ULN) for ALT is 30 IU/L in men and 19 IU/L in women [30]. The reported ALT levels in this review are more than twice the ULN and should be considered pathologic and require more workup [31]. It is suggested that ketamine hepatotoxicity can result in LFT derangements similar to that of drug-induced liver injury [32]. This effect can be augmented via inhibiting CYP32A enzyme, which is a hepatic enzyme responsible for ketamine breakdown [33], and is also demonstrated by an animal study [34].

The duration of ketamine abuse ranged from 3 months to 15 years, with a median of 2 years. The therapeutic dose for ketamine varies depending on its indication. Anesthesia, pain management, and depression require a dose of 1 mg/kg, 0.4 mg/kg, and 0.5 mg/kg, respectively [35–37]. The consumed ketamine dose in this review is patient-reported and was up to 3 g per day, which is substantially higher than the accumulated therapeutic dose at first glance, which is held responsible for causing hepatobiliary disturbances in this population. In the current review, all patients except one, who lacked outcome information, were discharged with improved symptoms and LFT results. This is in contrast with the disease outcome in ketamine-induced cholangiopathy in critically ill patients, which had a significant mortality rate [38]. This can be explained by the fact that the patients in this review did not have any underlying liver pathology and they were not in critical condition.

Imaging studies played a crucial role in the diagnosis and assessment of patients. The preferred method to visualize biliary tree was MRCP [39]. Specifically, in the current review, 13 patients underwent ultrasound, 9 underwent MRCP, 10 underwent CT, and 3 underwent HIDA scans. In our study, HIDA revealed diminished gall bladder ejection fraction in one patient indicative of functional gallbladder dysfunction [40], while two patients had normal results. The most common imaging finding was common bile duct (CBD) dilation, observed in 12 patients. Similar to the results of this review, a retrospective study showed that CBD dilation in ketamine-induced cholangiopathy can be found in up to 69% of patients [41].

The growing bulk of evidence regarding the etiology of these complications is not consistent. At a molecular level, ketamine can potentially contribute to biliary tract dilation by affecting NMDA receptors on smooth muscle cells [42]. Additionally, increased glutamate and NMDA concentration in dorsal motor nucleus of vagus nerve seem to increase gallbladder motility, which can be potentially antagonized by ketamine [20]. This may, in turn, lead to strictures in biliary tract, chronic inflammation, and fibrosis [21]. These conjectures of direct toxicity of ketamine are supported by several studies that were carried out to evaluate ketamine’s effect on urinary system [43, 44]. An animal study by Thune *et al*. suggests that ketamine stimulates opiate receptors and is associated with an increase in flow resistance through the sphincter of Oddi [45]. On the contrary, the results of sphincter of Oddi manometry in human subjects receiving low-dose ketamine for endoscopy does not support this claim [46].

This retrospective review was subject to several limitations. Due to the anecdotal nature of case reports, we could not draw robust conclusions. We used narrative synthesis for the most part and could not perform a meta-analysis due to the obvious heterogeneity. In addition, the number of patients who were selected was considerably low and several of them lacked relevant clinical data or were of low quality. We did not include retrospective observational studies that did not contain the details on individual patients. In spite of these limitations, this review used a systematic approach and endeavored to summarize the characteristics of biliary tract diseases in ketamine abusers.

## Conclusion

The current review provides important clinical information regarding the characteristics of patients with ketamine-induced cholangiopathy. These patients tend to be young men with deranged LFT and abdominal pain. Our study also highlights the diverse presentations and diagnostic modalities used in assessing these patients, emphasizing the importance of a multidisciplinary approach in their management.

## Data Availability

All the data regarding this manuscript is provided within the main document.

## Appendix

### Full search terms

((("secondary sclerosing cholangitis"[Title/Abstract] AND "case reports"[Publication Type]) OR ("cholestatic liver injury"[Title/Abstract] AND "case reports"[Publication Type]) OR ("biliary"[Title/Abstract] AND "case reports"[Publication Type]) OR ("Biliary Tract Diseases"[MeSH Terms] AND "case reports"[Publication Type]) OR ("Cholangitis"[MeSH Terms] AND "case reports"[Publication Type]) OR ("hepatobiliary disease"[Title/Abstract] AND "case reports"[Publication Type]) OR ("biliary dilatation"[Title/Abstract] AND "case reports"[Publication Type]) OR ("cholestasis"[Title/Abstract] AND "case reports"[Publication Type]) OR ("cholangitis, sclerosing"[MeSH Terms] AND "case reports"[Publication Type])) AND "case reports"[Publication Type] AND (("Ketamine"[Title/Abstract] OR "Ketamine"[MeSH Terms]) AND "case reports"[Publication Type])) AND (casereports[Filter]).
